# Lack of a site-specific phosphorylation of Presenilin 1 disrupts microglial gene networks and progenitors during development

**DOI:** 10.1371/journal.pone.0237773

**Published:** 2020-08-21

**Authors:** Jose Henrique Ledo, Ran Zhang, Luka Mesin, Diego Mourão-Sá, Estefania P. Azevedo, Olga G. Troyanskaya, Victor Bustos, Paul Greengard

**Affiliations:** 1 Laboratory of Molecular and Cellular Neuroscience, The Rockefeller University, New York, New York, United States of America; 2 Lewis Sigler Institute for Integrative Genomics, Princeton University, Princeton, New Jersey, United States of America; 3 Laboratory of Lymphocyte Dynamics, The Rockefeller University, New York, New York, United States of America; 4 Laboratory of Immune Cell Epigenetics and Signaling, The Rockefeller University, New York, New York, United States of America; 5 Laboratory of Molecular Genetics, The Rockefeller University, New York, New York, United States of America; 6 Flatiron Institute, Simons Foundation, New York, New York, United States of America; Institut d’Investigacions Biomediques de Barcelona, SPAIN

## Abstract

Microglial cells play a key role in brain homeostasis from development to adulthood. Here we show the involvement of a site-specific phosphorylation of Presenilin 1 (PS1) in microglial development. Profiles of microglia-specific transcripts in different temporal stages of development, combined with multiple systematic transcriptomic analysis and quantitative determination of microglia progenitors, indicate that the phosphorylation of PS1 at serine 367 is involved in the temporal dynamics of microglial development, specifically in the developing brain rudiment during embryonic microgliogenesis. We constructed a developing brain-specific microglial network to identify transcription factors linked to PS1 during development. Our data showed that PS1 functional connections appear through interaction hubs at *Pu*.*1*, *Irf8* and *Rela-p65* transcription factors. Finally, we showed that the total number of microglia progenitors was markedly reduced in the developing brain rudiment of embryos lacking PS1 phosphorylation compared to WT. Our work identifies a novel role for PS1 in microglial development.

## Introduction

Presenilin 1 *(Psen1* gene*–*PS1 protein) is the catalytic subunit of γ-secretase, an enzyme complex responsible for the cleavage of an extensive number substrates [1,2]. Particularly, γ-secretase is responsible for the conversion of the amyloid precursor protein (APP) into amyloid-β (Aβ) peptide [3]. PS1 has been most studied in the context of Alzheimer’s disease (AD), largely because mutations that cause early onset of AD are found most frequently in *Psen1* gene and PS1 also plays a key role in Aβ production. However, in addition to its key role in AD, PS1 plays a fundamental role in early brain development [2, 4]. PS1 is post-translationally regulated by phosphorylation of specific residues, including serine 367 which is conserved in humans [5,6]. Our previous reports showed that, upon phosphorylation of S367 by CK1γ, PS1 can decrease Aβ levels through an autophagy-mediated mechanism in neurons and through degradation of soluble Aβ by microglia [6–8]. Our previous work also showed that microglia containing PS1 S367A is functionally impaired [8]. Here we interrogated whether phosphorylation of PS1 at serine 367 plays a role in microglial development. We used multiple systematic transcriptomic analyses, novel bioinformatic approaches and quantitative determination of microglial progenitors, from embryonic brain development to adulthood mice lacking phosphorylation of PS1 at serine 367 compared to WT controls. Our data showed that PS1 S367A disrupts microglial gene networks and progenitors during development, suggesting a novel role for PS1.

## Results

Cellular function and lineage commitment can be affected by small changes in gene expression during development. Failure to acquire the correct cellular identity signature, commonly described as a set of expressed genes that gives a cell its unique character, might indicate a possible disruption in the developmental process. Establishment of microglial identity is shaped by the cell developmental ontogeny and its interaction with the environment [9]. During development, microglia lineage commitment initiates with migration of erythromyeloid progenitors from the yolk sac to the brain rudiment at embryonic days 9.5–10.5 (E9.5–10.5), where they develop and mature under three distinct temporal stages, namely early (E10.5−E14), pre- (E14−P9) and adult (4 weeks and onwards) microglia [10,11].

**Fig 1:**
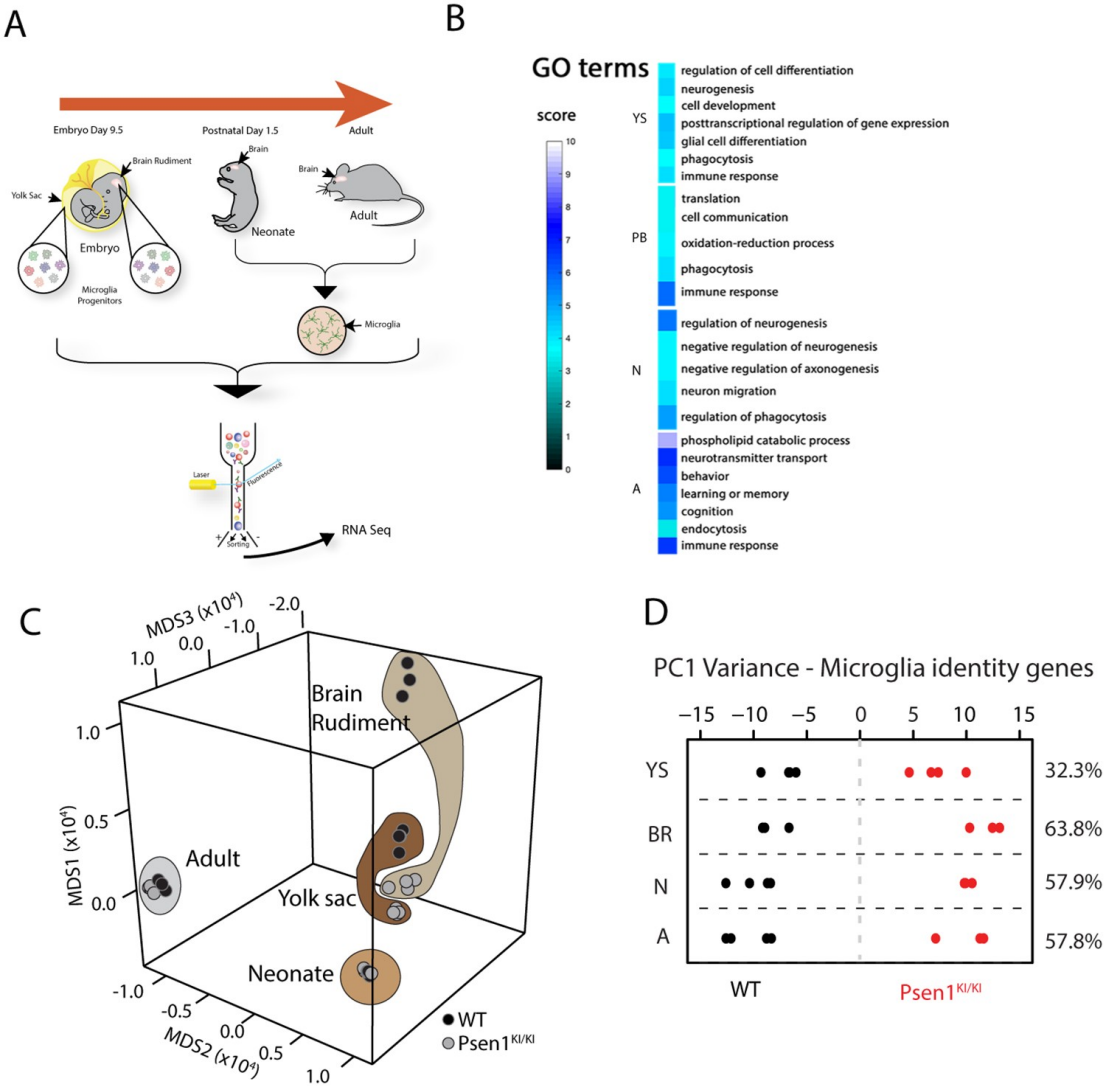
Gene-expression patterns of early, pre- and mature WT and Psen1^KI/KI^ microglia. RNA sequencing performed in microglia progenitors from yolk sac and brain rudiment at embryonic day 9.5, pre-microglia at postnatal day 1.5 and adult microglia at 3 months. (A) Schematic diagram showing the strategy used to profile microglia-enriched transcripts during key periods of development. (B) Gene Set Enrichment Analysis (for details see Methods) was used to identify GO Biological Processes differentially regulated in Psen1^KI/KI^ microglia (C) Multidimensional scaling of all genes of microglia progenitors in yolk sac, brain rudiment and pre-adult and adult microglia. Significance of difference between WT and Psen1^KI/KI^ microglia between developmental stages are assessed by permutation test (for details see Methods). (D) Principal component analysis restricted to microglia identity genes expressed in WT or Psen1^KI/KI^ early, pre- and adult microglia (YS = Yolk Sac, BR = Brain Rudiment, N = Neonate, A = Adult). For microglia progenitors, N = 3–4 biologically independent measures from 6–12 pooled yolk sacs or brain rudiment. For neonate and adult microglia, N = 4 animals per group.

We systematically profile microglia-specific transcripts during multiple key development time points. To profile microglia-specific transcripts from *Psen1 S367A* (denoted, *Psen1*^*KI/KI*^) mice and WT littermate controls, we sorted early microglial progenitors from the yolk sac (YS; E9.5) and brain rudiment (BR; E9.5), pre-microglia from P1.5 neonates (N) and mature microglia from adult mice (A; 12 weeks) (Fig 1A, S1 Fig). Firstly, we assessed processes and pathways affected by the *Psen1*^*KI/KI*^ mutation compared WT controls through GO biological process enrichment analysis (Fig 1B). Considering all microglial developmental stages analyzed, GO enrichment revealed that the most common processes impaired in *Psen1*^*KI/KI*^ microglia compared to control microglia are related to cell differentiation, phagocytosis and immune response (Fig 1B). Specifically, in yolk sac (YS), the GO analysis showed a decrease enrichment in pathways related to cell development and differentiation in *Psen1*^*KI/KI*^ early-microglia progenitors compared to WT controls (Fig 1B). Interestingly, we also observed alterations in pathways related to immune response, phagocytosis and behavior in the *Psen1*^*KI/KI*^ microglia from brain rudiment, neonates, and adults compared to WT controls (Fig 1B). We next assessed the effect of the *Psen1*^KI/KI^ across developmental stages by applying multidimensional scaling (MDS) as a means to graphically represent relative distance between experimental samples in a three-dimensional Euclidean space, where shorter distance between points reflects greater similarity. This analysis demonstrated a robust difference between WT and *Psen1*^KI/KI^ microglia progenitors in the brain rudiment (Fig 1C). Notably, distance between WT vs *Psen1*^KI/KI^ microglia progenitors in the brain rudiment is significantly greater than the distance between WT vs *Psen1*^KI/KI^ microglia progenitors in the yolk sac or neonate or adult stages (p ≤ 0.001, Fig 1C).

We next examined transcriptional patterns of WT and *Psen1*^*KI/KI*^ microglia using PCA analysis restricted to microglia identity signature genes. Microglia identity signature genes defined as 239 genes that are specifically expressed in mouse microglia compared to monocytes and other immune cell types [12]. The data showed a clear separation between WT and *Psen1*^*KI/KI*^ microglia from early development to adulthood. (Fig 1D).

**Fig 2:**
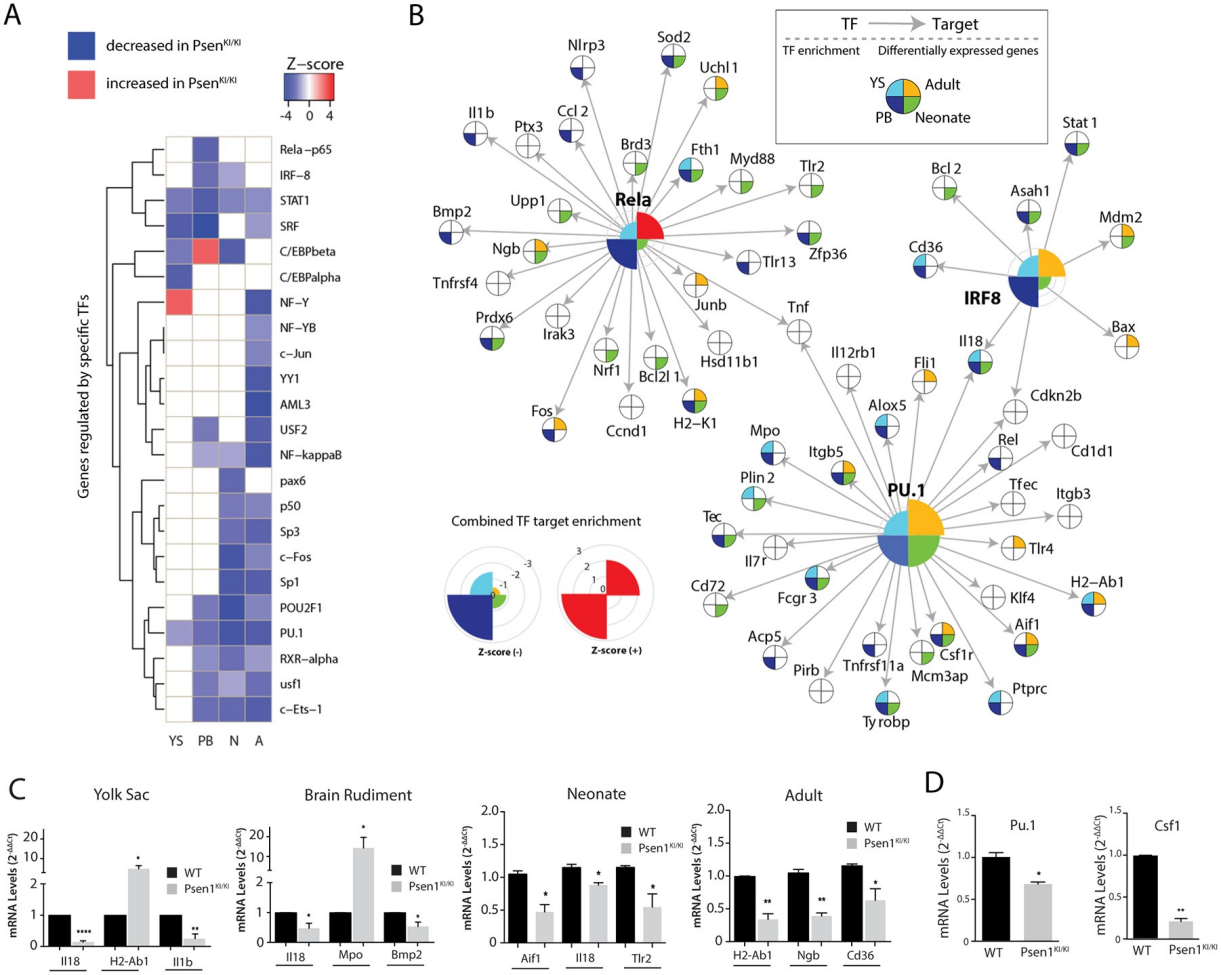
Key transcription factors in different microglia developmental stages and their functional association with Psen1. (A) Identification of differentially expressed TF targets across microglia development. Gene targets regulated by TFs experimentally annotated in TRANSFAC (detailed in Methods), Z-scores heatmap of the gene targets of 18 TFs (YS = Yolk Sac, BR = Brain Rudiment, N = Neonate, A = Adult). (B) Gene targets regulated by Irf8, Pu.1 and Rela according to experimental annotations in TRANSFAC database (detailed in the Methods section). Targets differentially expressed in Psen1^KI/KI^ vs WT (FDR<0.1) are color-coded with respect to each developmental stage where such differential expression is observed. (C) Quantitative RT-PCR of the most differentially expressed TFs targets in Psen1KI/KI vs WT. Pu.1 targets are H2-Ab1, Mpo, Aif1 and H2-Ab1 (YS, BR, Neonate and Adult respectively), Irf8 targets Il18 (YS, BR and Neonate) and Cd36 (adult), Rela targets Il1b, Bmp2, Tlr2 and Ngb. (YS, BR, Neonate and Adult respectively). (D) Quantitative RT-PCR of Pu.1 and Csf1r in microglia from brain rudiment. Bar plots represent means ± sem. *p < 0.05, **p < 0.01, ****p < 0.0001, 2 sample t-test. For microglia progenitors, N = 3–4 biologically independent measures from 6–12 pooled yolk sacs or brain rudiment. For neonate and adult microglia, N = 4 animals per group.

To gain further insights into the TFs that regulate key gene networks disrupted in *Psen1*^KI/KI^ microglia development, we identified TFs regulators of microglia development and function based on the experimental TRANSFAC annotations database on transcription factors, their binding sites and regulated genes [13]. Interestingly, targets of Pu.1, a TF critically involved in microglia development (Fig 2A) [10,14], were differentially expressed in all microglial developmental stages. Targets of Irf8, a TF also known to play an important role in microglia development [14], were differentially expressed mostly in the brain rudiment microglial progenitors and neonate microglia (Fig 2A), suggesting a specific role for this TF at these periods of development. Notably, the TF Rela−p65 (NF-kappa-B p65 subunit protein encoded by the Rela gene) also showed its gene targets differentially expressed mostly in brain rudiment microglial progenitors, suggesting a specific role for it during these periods of development (Fig 2A). Further analysis of these essential microglial TFs across developmental periods showed that many of their gene targets are differentially expressed in *Psen1*^*KI/KI*^ compared to WT microglia (15% of gene targets are differentially expressed in yolk sac, 41% in brain rudiment, 33% in neonate, and 21% in adult) (Fig 2B). We next validated the most differentially expressed Pu.1, Irf8 and Rela targets by qPCR. We confirmed that the most differentially expressed genes described in our bioinformatics analysis of RNAseq were altered by *Psen1*^*KI/KI*^ (Fig 2C). Due to the critical importance of Pu.1 for microglial development we also investigated Pu.1 expression levels in *Psen1*^KI/KI^ microglia compared to WT. Indeed, mRNA levels of Pu.1 was decreased in *Psen1*^*KI/KI*^ microglia compared to WT microglia (Fig 2D).

**Fig 3:**
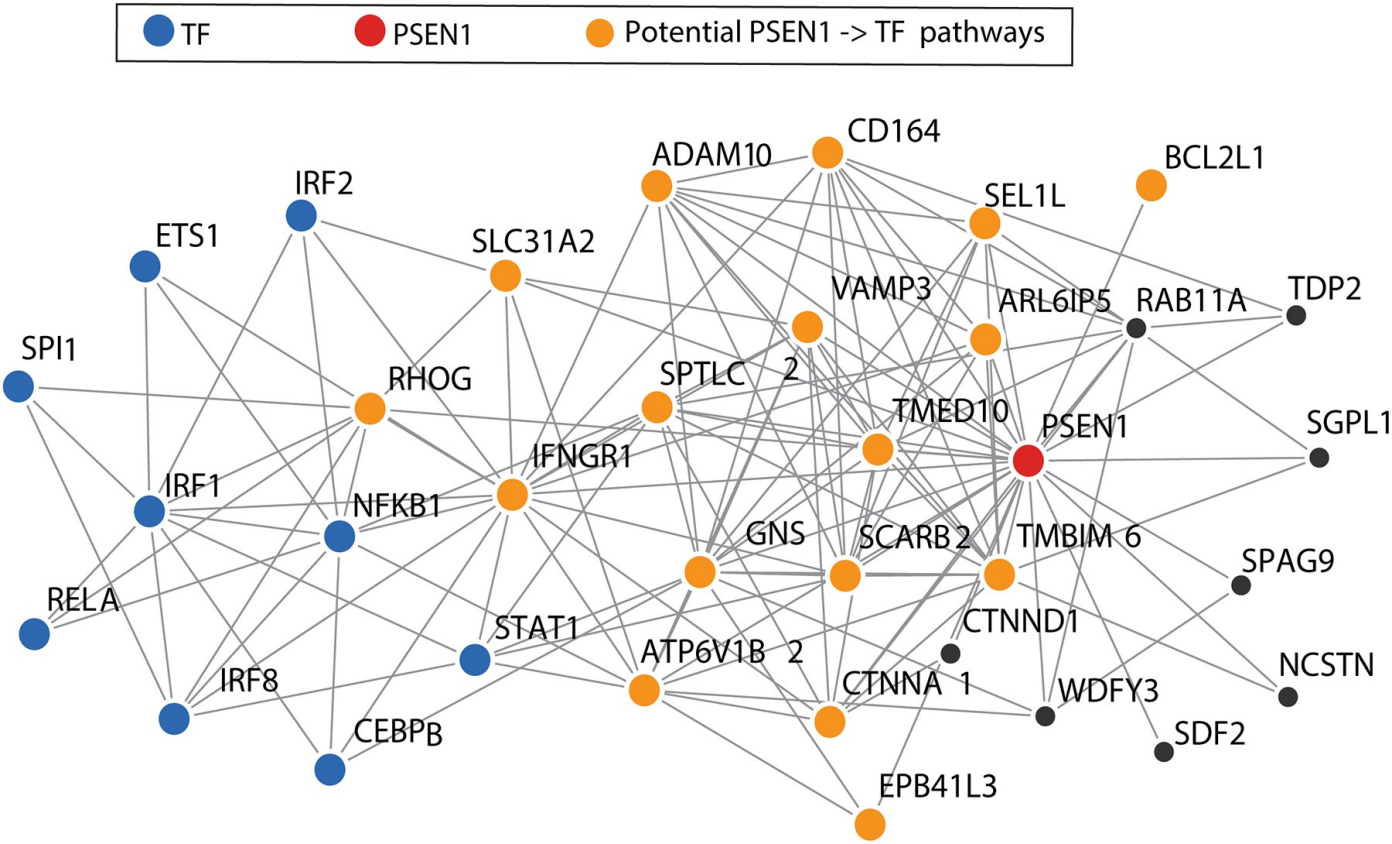
Developing brain-specific microglial functional network. Developing brain-specific microglial functional network (for details see Methods) around Psen1 (red circle). Top 25 genes (orange and black circles) most closely associated with Psen1 are shown in the network. Genes connected to the TFs (orange circles) in no more than 2 steps are represented as the potential genes linking Psen1 to the TF. Only strong functional interactions (five-fold over prior) are shown in the plot.

To better understand the functional connections of PS1 to the transcription factors affected by *Psen1*^*KI/KI*^ in the developing brain rudiment (E9.5) and to gain insights into functional genomic aspects of developing microglia in mice and humans, we constructed for the first time a developing brain-specific microglial functional network using an innovative approach presented in Greene et al., (2015) [15,16]. This approach gives us the advantage of investigating aspects of microglia in early development where human dataset is inexistent. The network represents a functional gene interaction map of microglia built through probabilistic integration of a large compendium of human functional genomic data (including thousands of expression, physical interaction, and transcriptional regulation datasets) using microglia-specific progenitor genes from our sequencing data (S1 Table) and Butovsky et al. [12] to provide the microglia-specific context. Intuitively, the mouse microglia-specific genes serve as a “hook” to extract microglia-relevant signals from the human public data collection. Exploiting this developing brain rudiment microglial network, we identified, from within the 25 most functionally linked PS1 neighbors (orange and black circles), the potential candidate genes potentially mediating the functional connection between PS1 and the identified TFs (Fig 3, orange circles).

**Fig 4:**
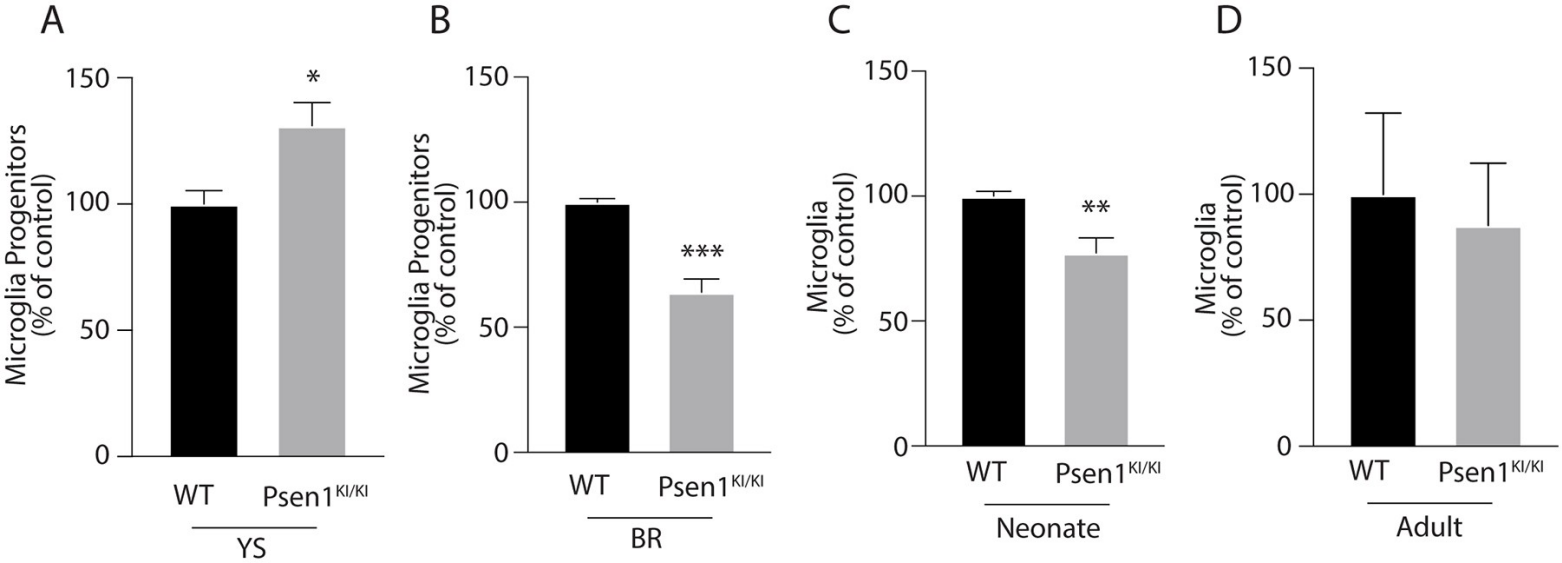
Psen1^KI/KI^ disturbs microglial progenitors. Number of microglia or progenitors measured by flow cytometry in yolk sac (A), brain rudiment (B), neonates (C) and adult (D). Bar plots represent means ± sem. *p < 0.05, ****p < 0.0001, 2 sample t-test. For microglia progenitors, N = 3–4 biologically independent measures from 6–12 pooled yolk sacs or brain rudiment. For neonate and adult microglia, N = 3–4 animals per group.

During development, disrupting gene expression or silencing programs may affect proliferation, migration, fate decisions and survival, all of which collectively dictate progenitor cell number [17,18]. Thus, we sought to investigate the number of microglia from *Psen1*^*KI/KI*^ and WT control mice. We analyzed the number of microglia progenitors in the yolk sac, brain rudiment, pre-microglia in the neonate brain and adult microglia (Fig 4). We found that the number of microglial progenitors in the yolk sac was significantly increased in *Psen1*^*KI/KI*^ mice compared WT controls (Fig 4A). A marked reduction in *Psen1*^KI/KI^ microglial progenitors was observed in the developing brain rudiment (Fig 4B) and in pre-microglia in neonates (Fig 4C) compared to WT controls. We did not observe a difference in microglial number in adult *Psen1*^KI/KI^ mice compared to WT controls (Fig 4D), as shown in our previous report [8].

## Discussion and conclusion

Using the experimentally annotated TRANSFAC database to identify TFs that control the transcriptional changes observed through *Psen1*^KI/KI^ microglial development, we identified TFs that regulate a set of genes differentially expressed in *Psen1*^KI/KI^ microglia in different microglial developmental stages. Among them, we identified *Pu*.*1*, a TF necessary for proper microglia development [10]. Moreover, gene targets of *Irf8*, another TF that plays a central role in microglial development [14], were differentially expressed only in brain rudiment and neonatal stages, suggesting that *Psen1* may play a specific role at this period of development, acting through *Pu*.*1* and *Irf8*. Pu.1 seemed to be involved in regulating transcription of genes in *Psen1*^KI/KI^ microglia also in the adult brain, suggesting that they might have distinct roles both in microglia development early maturation and during adulthood. For instance, *Pu*.*1* is considered a master regulator of microglial development [10] and has been implicated in Aβ phagocytosis in human adult microglia [19]. Importantly, *Pu*.*1* is considered an essential lineage-determining transcription factor that selects enhancers by interacting with other TFs [20]. For example, Pu.1 occupies enhancer landscapes and can act in a collaborative or hierarchical manner with signal-dependent transcription factors such as NF-kappa-B [21,22]. Interestingly, we identified *Rela-p65* (NF-kappa-B p65 subunit) as the TF in which gene targets are differentially expressed mostly in microglial progenitors in the developing brain rudiment, suggesting it plays an exclusive role in early stages of microglia development. This link is further supported by prior observations that hematopoietic *Rela-p65*-null mice show defective self-renewal and differentiation of hematopoietic stem and progenitor cells [23]. Based on the evidence above, we speculate that *Psen1* may act through Pu.1 and Rela-p65 and contribute to lineage determination in microglial progenitors. The exact mechanism connecting *Psen1* to *Pu*.*1* and *Rela-p65* requires further investigation. Intriguingly, genes such Stat1 was affected in all developmental stages we had analyzed, while others such as Fli1 was affected in the adult microglia only. Fli1 is a transcription factor found to play an important role in in hematopoietic stem/progenitor maintenance and proliferation [24–26]. Fli1 and Pu.1 are members of the ETS transcription factor family and Pu.1 is a positive regulator of Fli1 gene [27]. Thus, it would be interesting to investigate whether the Pu.1 = > Fli1 axis is essential for adult microglia proliferation and maintenance in future studies.

Our study also demonstrated that *Psen1*^KI/KI^ microglia maintain a distinct molecular identity signature compared to WT microglia from development to adulthood. Interestingly, the total number of microglia progenitors was markedly reduced in the developing brain rudiment of *Psen1*^KI/KI^ embryos compared to WT, which could be explained by defective migration from the yolk sac, proliferation, cell fate decisions or survival. Although the mechanisms by which *Psen1* regulates the microglial gene networks and number of progenitors during development remain to be determined, GO enrichment and our data on the functional network of developing brain rudiment microglia point to the Interferon-γ receptor as a possible target mediating those effects. Interestingly, Interferon-γ has been shown to act as an anti-proliferative molecule in different cell types [28].

Our bioinformatics analysis suggests that PS1 might act on Pu.1 and IRF8 through interaction hubs at TMED10, RHOG and IFNGR1. TMED10 gene encodes a transmembrane protein (TMP21) that has multiple roles within the cell that extend beyond its classical role in vesicle trafficking. TMP21 is shown to interact with PS1 to regulate γ-secretase activity [29]. TMP21 is also shown to disrupt TGF-β complex formation which attenuates TGF-β signaling [30]. TGF-β is a known regulator of cellular development, differentiation and Pu.1 induction in cell progenitors [31]. Moreover, TGF-β is required for microglia unique gene expression signature [12]. Thus, a potential signaling pathway by which PS1 regulates Pu.1 might be via Tmed10 = > TGF-β.

Future studies are required to a better understanding of the underlying molecular mechanisms linking PS1 to microglial development. Nonetheless, our data indicate a novel role of PS1 in the dynamics of microglial development.

## Materials and methods

### Mice

C57BL/6 (000664) mice were purchased from the Jackson Laboratories and maintained in our facilities. *Psen1*^KI/KI^ constitutive knock-in C57BL/6J mice were generated by homologous recombination targeting exon 10. *Psen1*^KI/KI^ mice were maintained and crossed as homozygous and WT littermate controls were used for experiments. Genotyping was carried out by Transnetyx.

All mice were maintained at The Rockefeller University Animal facilities and used at 12 weeks of age (adult). Both female and male mice were used for experiments. Animal care and experimentation were according to NIH guidelines and were approved by the Institutional Animal Care and Use Committee at The Rockefeller University (protocol #18035H). Mice were euthanized via cervical dislocation under isoflurane anesthesia. All efforts were made to minimize animal suffering.

### Sample preparation and flow cytometry analysis

Adult mice were anesthetized with a ketamine/xylazine cocktail and perfused with 25 ml of Ca2+/Mg2+-free DPBS (Sigma) prior to brain collection. Neonate (P1.5) brains were collected after decapitation. Yolk sac and developing brain rudiment were collected from embryos at developmental stage E9.5. Yolk sac, brain rudiment and whole brains were collected and placed in FACS buffer (PBS containing 5% FBS and 10 mM HEPES) at 4°C. Samples were minced with scissors and incubated with 4000 U/mL of collagenase D (Roche, 11088858001) at 37 °C for 30 min. Collagenase was inactivated by adding 10 mM EDTA for an additional 5-min incubation at 37 °C. Digested material was passed through a 70 μm cell strainer. Cells were then washed and centrifuged at 2,000 r.p.m. in 38% Percoll gradient for 30 min. Cell pellets were washed and resuspended in FACS buffer. Nonspecific binding to FC receptors was blocked by incubation with a CD16- and CD32-specific FC blocking antibody (BD-Pharmingen 553141) for 15 min. Cells were washed and stained with the antibody against specific molecular markers to certify cell population specificity according to Kierdorf et al [14], below. Number of microglia/ progenitors was calculated as a percentage of live cells.

Yolk Sac and Brain Rudiment: CD45^HIGH^–Cx3cr1^+^–F4/80^HIGH^–cKit (negative).
Neonate and Adult: CD45^INT^–Cx3cr1^+^–F4/80^+^–Cd11b^+^.

Fluorescent-dye-conjugated antibodies were purchased from Biolegend (anti-CD115 (Csfr1), 135510; anti-F4/80, 123131; anti-Cx3Cr1, 149016; anti-CD117 (c-kit), 105824; anti-Ly6c, 128033), Invitrogen (Anti-CD11b, 47-0112-82; anti-CD45, 56-0451-82) or BD-Pharmingen (anti-CD16/CD32 (FC blocking); 553141). Live cells were verified using DAPI (4’,6-Diamidino-2-Phenylindole, Dilactate) or Aqua Dead Cell Stain kit (L34957, Invitrogen), (D3571, Thermofisher). Antibodies were used 1:400, except for anti-CD45 (1:150) and F4/80 (1:200). Samples were incubated with antibodies for 30 min at 4°C. FACS sorting was performed using an ARIA II sorter using an 85-μm nozzle (Becton Dickinson). Flow cytometry data were analyzed using FlowJo software (Tree Star).

### RNA sequencing and bioinformatics

#### Yolk sac and brain rudiment microglia progenitors

200–400 cells per each sample were sorted directly into TCL lysis buffer (Qiagen, 1031576) supplemented with 1% β-mercaptoethanol followed by RNA isolation with Agencourt RNAClean XP Beads (Beckman Coulter, A63987). RNA was then reverse transcribed into cDNA using an oligo(dT) primer and amplified as described [32]. Nextera XT (Illumina, FC-131-1024, FC-131-1001) was used to prepare a multiplexed pooled library of fragmented and uniquely indexed samples for each flow-sorted population. Single reads 150 bp sequencing was performed on a NextSeq 500 (Illumina).

#### Neonate and adult microglia

20000 cells per each sample were sorted directly into of RLT buffer (provided by the manufacturer) containing DTT. After, RNA was removed from the beads with a magnet. Homogenized lysate was then transferred to a gDNA eliminator spin column placed in a 2 ml collection tube (supplied by the manufacturer). The following steps were conducted according to the instructions provided by the manufacturer. For all RNA samples, RNA integrity number (RIN) was ≥ 8.5.

After RNA isolation, 1 ng of total RNA was used to generate full length cDNA using Clontech’s SMART-Seq v4 Ultra Low Input RNA Kit (634888). cDNA was then used to prepare libraries using Illumina Nextera XT DNA sample preparation kit (FC-131-1024). Libraries with unique barcodes were pooled at equal molar ratios and sequenced on Illumina NextSeq 500 sequencer to generate 150 bp single reads, following manufacturer’s protocol.

The reads were aligned using the STAR version 2.3.0 software [33] that permits unique alignments to Mouse Ensembl genes. Differential expression was determined using edgeR software [34] with default settings. Expression is given in Counts per million (CPM).

Gene Set Enrichment Analysis (PAGE: Parametric Analysis of Gene Set Enrichment—[35]) of differentially expressed genes was performed to identify enrichment of curated gene sets derived from the Gene Ontology Biological Processes pathway (GObp) database, GO terms with 10–500 in size were used in our analysis. The rank score was determined by log of p-value, with symbols indicating up/down regulation. All GO terms selected are with significance FDR ≤ 0.01.

GO enrichment analysis according to Gene Ontology Resource updated in 06-01-2020.

#### Microglia identity signature genes

Defined as 239 genes that are specifically expressed in mouse microglia compared to monocytes and other immune cell types [12]. The six microglial signature genes evaluated in this study were selected from the 239 microglial signature identity genes identified in previously [12].

#### Brain rudiment and yolk sac developmental separation

3D Multidimensional scaling plot was generated with Euclidean distance. To check for sample separation, we performed variance stabilization transformation by DESeq [36] to remove potential dependence of variance on the mean. The statistical test was performed based on the mean Euclidean distance between brain rudiment, WT versus *Psen1*^KI/KI^ and *Psen1*^KI/KI^ versus WT in yolk sac or neonate or adult. The p-value was estimated by permuting gene expression across samples by gene 1000 times and calculating the likelihood of the test statistic greater or equal to the observed one. To rule out the possibility of single/several genes driving the separation, we randomly sampled 5000 genes out of the approximately 20000 total genes for 100 times to ensure of the robustness. For the six-genes referred to as microglia identity signatures, to assess the separation in brain rudiment WT versus *Psen1*^KI/KI^ and yolk sac WT versus brain rudiment *Psen1*^KI/KI^, similar permutation test was conducted without subsampling due to limited gene set size. Permutation p-values were according to Phipson and Smyth [37].

#### Transcriptional factors targets identification

We used the TRANSFAC [13] mouse database to acquire a curated set of experimentally transcriptional factor targets in mouse. Parametric analysis of gene set enrichment (PAGE) [35] was used to get enrichment z-score for TF targets.

#### Network analysis and construction of developing microglia-specific functional network

A human brain rudiment microglia network was constructed according to [15] to represent the functional linkage between each pair of genes in brain rudiment microglia. We trained a naïve Bayesian classifier to predict the functional linkage between each pair of genes in the genome by integrating thousands of large-scale datasets (datasets additional to [15] in S1 Table). Positive gold standards were gene pairs both of which are expressed in microglia brain rudiment and have known functional interactions, while negatives were pairs either not co-expressed in brain rudiment or not functionally connected. Specifically, brain rudiment microglia expressed genes were determined by the intercept of genes highly expressed (CPM>50) in brain rudiment microglia samples and lowly expressed (CPM<10) in adult microglial samples in the data presented in this study, as well as genes highly expressed in microglia compared to neurons, oligodendrocytes, astrocytes, or compared to myeloid and other immune cells according to [12]. The network was evaluated by cross validation, as well as the ability to recapitulate differentially expressed genes in *Psen1*^KI/KI^ by *Psen1* neighbors in the network. Human genes were mapped back to mouse based on the combination of sequence and functional conservation using the Functional Knowledge Transfer (FKT) approach [38].

### Real-time relative quantification PCR

Taqman reagents were used for qPCR. Actin Beta (ActB) was used to normalize samples. Predesigned probes used were purchased from IDTDNA. mRNA levels are expressed using the 2^−ΔΔCt^ method [39].

## Supporting information

S1 Fig FACS gating strategy for isolating progenitors of microglia or neonate and adult microglia. Yolk sacs or developing brains (brain rudiment) were harvested from WT or *Psen1*^KI/KI^ mice days E9.5 and gated for CD45^HIGH^, F4/80^HIGH^, CX3CR1^+^ and c-kit^-^. Brains were harvested from WT or *Psen1*^KI/KI^ mice at postnatal day 1.5 or adult (12 weeks) gated for CD45^INT^, F4/80^+^, CX3CR1^+^, Cd11b^+^. N = 3–4 biologically independent measures from 6–12 pooled yolk sacs or developing brains (brain rudiment). For neonate and adult experiments, N = 3–4 individual brains per group.
(TIF)

S1 Table (XLSX)
