# Correction: Lack of a site-specific phosphorylation of Presenilin 1 disrupts microglial gene networks and progenitors during development

**DOI:** 10.1371/journal.pone.0247680

**Published:** 2021-02-19

**Authors:** Jose Henrique Ledo, Ran Zhang, Luka Mesin, Diego Mourão-Sá, Estefania P. Azevedo, Hernandez Moura Silva, Olga G. Troyanskaya, Victor Bustos, Paul Greengard

Hernandez Moura Silva should be included in the author byline. Hernandez Moura Silva should be listed as the sixth author, and his affiliation, which is not indicated, is 7: Kimmel Center for Biology and Medicine at the Skirball Institute, New York University Grossman School of Medicine, New York, 10016, United States of America. The contributions of this author are as follows: Conceptualization, data curation, formal analysis, investigation, methodology, project administration, software, supervision, validation, visualization, writing—review & editing.

The correct citation is: Ledo JH, Zhang R, Mesin L, Mourão-Sá D, Azevedo EP, Silva HM, et al. (2020) Lack of a site-specific phosphorylation of Presenilin 1 disrupts microglial gene networks and progenitors during development. PLoS ONE 15(8): e0237773. https://doi.org/10.1371/journal.pone.0237773
