# Supplementary figures and images for: Lack of a site-specific phosphorylation of Presenilin 1 disrupts microglial gene networks and progenitors during development
Source: PLoS One. 2020 Aug 21;15(8):e0237773. doi: 10.1371/journal.pone.0237773 (PMC7444478; doi:10.1371/journal.pone.0237773)

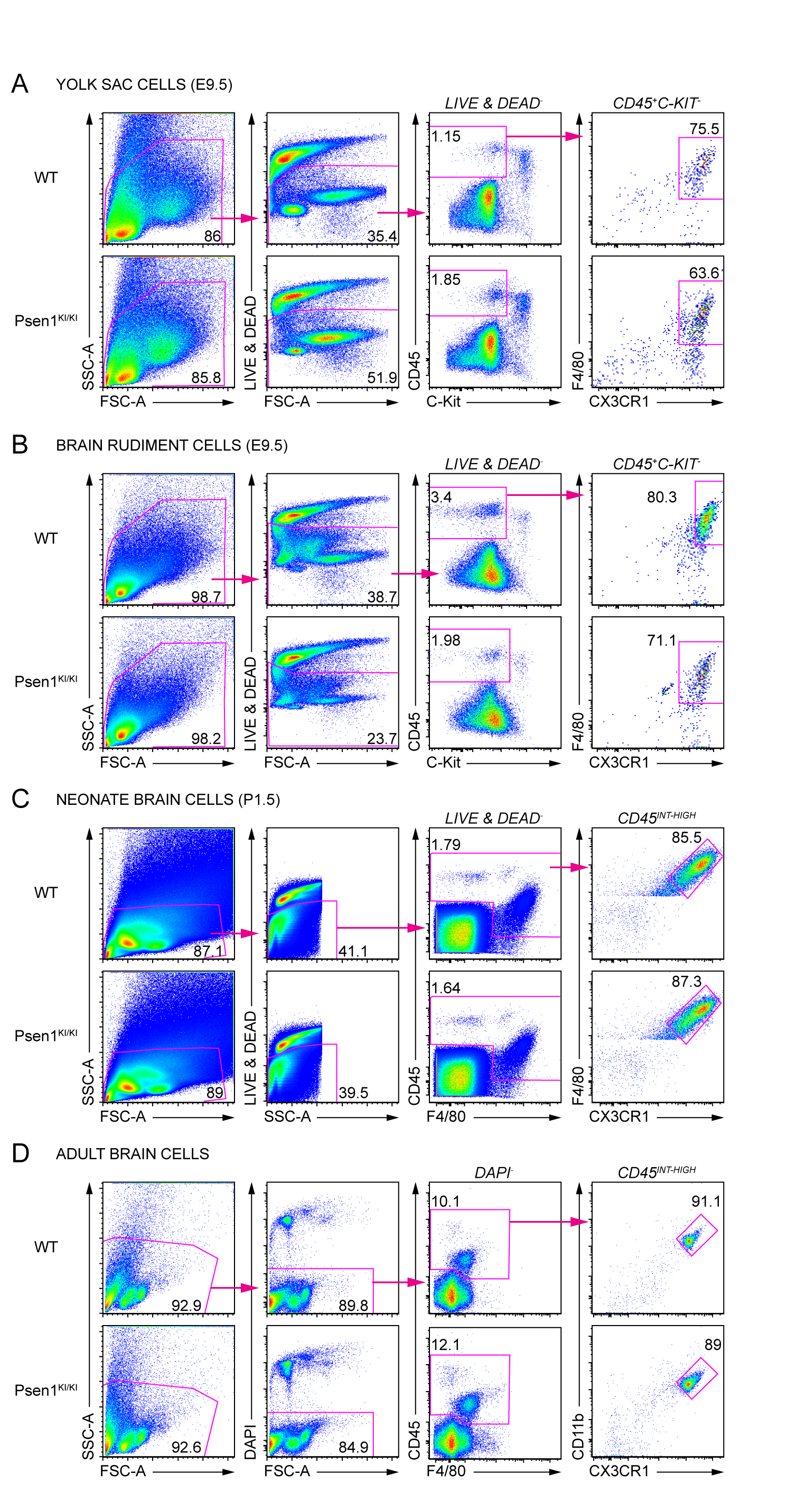

Supplement: S1 Fig — Yolk sacs or developing brains (brain rudiment) were harvested from WT or Psen1KI/KI mice days E9.5 and gated for CD45HIGH, F4/80HIGH, CX3CR1+ and c-kit-. Brains were harvested from WT or Psen1KI/KI mice at postnatal day 1.5 or adult (12 weeks) gated for CD45INT, F4/80+, CX3CR1+, Cd11b+. N = 3–4 biologically independent measures from 6–12 pooled yolk sacs or developing brains (brain rudiment). For neonate and adult experiments, N = 3–4 individual brains per group. (TIF) [file pone.0237773.s001.tif]
